# Mechanobiology of Autophagy: The Unexplored Side of Cancer

**DOI:** 10.3389/fonc.2021.632956

**Published:** 2021-02-26

**Authors:** Maria Paz Hernández-Cáceres, Leslie Munoz, Javiera M. Pradenas, Francisco Pena, Pablo Lagos, Pablo Aceiton, Gareth I. Owen, Eugenia Morselli, Alfredo Criollo, Andrea Ravasio, Cristina Bertocchi

**Affiliations:** ^1^ Laboratory of Autophagy and Metabolism, Department of Physiology, Faculty of Biological Sciences, Pontificia Universidad Católica De Chile, Santiago, Chile; ^2^ Laboratory for Mechanobiology of Transforming Systems, Institute for Biological and Medical Engineering, Schools of Engineering, Medicine and Biological Sciences, Pontificia Universidad Católica de Chile, Santiago, Chile; ^3^ Laboratory for Molecular Mechanics of Cell Adhesion, Department of Physiology, Faculty of Biological Sciences, Pontificia Universidad Católica De Chile, Santiago, Chile; ^4^ Advanced Center for Chronic Diseases (ACCDiS), Santiago, Chile; ^5^ Laboratory of Investigation in Oncology, Faculty of Biological Sciences Pontificia Universidad Católica de Chile, Santiago, Chile; ^6^ Millennium Institute on Immunology and Immunotherapy, Santiago, Chile; ^7^ Autophagy Research Center, Santiago de Chile, Chile; ^8^ Facultad De Odontología, Instituto De Investigación En Ciencias Odontológicas (ICOD), Universidad De Chile, Santiago, Chile

**Keywords:** autophagosome, biomembranes, cytoskeleton, mechanosensing, mechanotransduction

## Abstract

Proper execution of cellular function, maintenance of cellular homeostasis and cell survival depend on functional integration of cellular processes and correct orchestration of cellular responses to stresses. Cancer transformation is a common negative consequence of mismanagement of coordinated response by the cell. In this scenario, by maintaining the balance among synthesis, degradation, and recycling of cytosolic components including proteins, lipids, and organelles the process of autophagy plays a central role. Several environmental stresses activate autophagy, among those hypoxia, DNA damage, inflammation, and metabolic challenges such as starvation. In addition to these chemical challenges, there is a requirement for cells to cope with mechanical stresses stemming from their microenvironment. Cells accomplish this task by activating an intrinsic mechanical response mediated by cytoskeleton active processes and through mechanosensitive protein complexes which interface the cells with their mechano-environment. Despite autophagy and cell mechanics being known to play crucial transforming roles during oncogenesis and malignant progression their interplay is largely overlooked. In this review, we highlight the role of physical forces in autophagy regulation and their potential implications in both physiological as well as pathological conditions. By taking a mechanical perspective, we wish to stimulate novel questions to further the investigation of the mechanical requirements of autophagy and appreciate the extent to which mechanical signals affect this process.

## Introduction

At its completion in 2003, the Human Genome Project (1) was saluted as the tool to finally cure every cancer. Two decades later, this largely anticipated promise has yet to be delivered and the community of cancer researchers, which was once disproportionally focused on the central dogma of molecular biology, now embraces more holistic views. One of the most exciting frontiers of cancer research deals with understanding homeostatic processes during cancer development and how cancer cells respond to environmental stresses of a chemical and physical nature. In this context, the catabolic activity of autophagy is the key mechanism to maintain the balance between synthesis, degradation, and recycling of cytosolic components (2). These routine housekeeping functions provide a cellular mechanism to preserve homeostasis, enhance resilience to stresses and promote cell survival. Several environmental stresses activate autophagy, among those hypoxia, DNA damage, inflammation, and metabolic challenges such as starvation. Aside from challenges of chemical nature, cells are also exposed to stresses of mechanical nature, arising from environmental cues. Sensing of mechanical stress (mechanosensing) is mediated by force-induced conformational changes of mechanosensitive proteins directly or indirectly connected to the cytoskeleton and by mechanically activated ion channels (3, 4). Mechanosensing results in a modification of intracellular tension through reorganization of cytoskeletal and actomyosin contraction, which, in turn, integrate the mechanical signals into biochemical cascades (mechanotransduction), and, at longer time scale, lead to modification of gene expression (4). Hence, the physical properties of the microenvironment, such as extracellular matrix composition, stiffness, and architecture, have a profound impact on cellular genotype, phenotype, processes, tissue organization and overall biological function of the organism (4). This relation between mechanics and biological responses is also important during cancer transformation and progression, where the specific physical microenvironment of the tumor cells undergoes dramatic changes. These modifications of the tumor microniche are driven by enhanced cell contractility, increased pressure resulting from abnormal cell proliferation and growth of tumor mass, and alterations of composition, architecture and rheological properties of the surrounding extracellular matrix (5, 6). It has been reported that these mechanical changes correlate with activation of autophagy, which may be part of an integrated response to mechanical stresses employed by cancer cells to escape programmed cell death and to facilitate their adaptative response to the new mechanical environment (7). Furthermore, compelling evidence have suggested that autophagy impact several cancer hallmarks including cell motility and invasion, cancer stem cell viability and differentiation, epithelial to mesenchymal transition (EMT), resistance to apoptosis and anoikis, escape from immune surveillance and tumor cell dormancy (7, 8). However, the causative relation between cellular mechanics and autophagy and their interdependent role in cancer transformation are fragmentary and largely anecdotical. Here, we aim to review the autophagic process using a mechanical perspective and explore the crosstalk between mechanotransduction and cellular catabolism in order to access their possible contribution to cancer transformation and survival.

## Role of Cytoskeleton in Cell Mechanics

Vital functions of eukaryotic cells such as resistance to deformation, control of cellular shape, migration and transport of intracellular cargos depend on the activity of the cytoskeleton, an interconnected network of filamentous polymers, motor proteins and regulatory proteins (9). This network is composed by three interdependent structural components, namely microtubules, intermediate filaments, and microfilaments (actin) which are the engine of the cells as they convert chemical energy into mechanical energy *via* ATP-dependent polymerization and action of motor proteins. This mechanical energy is used to produce forces that displace cellular elements (e.g. formation of cellular protrusion, transport of cargos) and/or store elastic energy therein (e.g. cortical tension, cellular contractility). The whole process of autophagy being a sequence of membrane remodeling events is mechanically accomplished and coordinated by ATP-dependent cytoskeletal dynamics that lead to mechanical deformation and transport (10, 11). The cytoskeleton acts as an important framework for the modulation and control of correct positioning, tethering, docking, priming, fusion, and movement of organelles, such as autophagosomes and lysosomes. Actin cytoskeleton is composed by actin filaments and fibers whose assembly and disassembly generate web-like networks (Arp2/3-mediated branching) and bundles (formin-dependent crosslinking of filaments). These networks and bundles structurally support cellular membranes and determine their dynamics (12). Importantly, the action of molecular motors of the myosin family puts actin filaments under tension. Similar to a stretch coil, the release of this tension produces kinetic energy used for vesicle transport and membrane remodeling associated to autophagosome formation (13, 14). In addition, some myosins [i.e. myosin VI (15)] are directly involved in the transport of various cargos including autophagosomes (15). Furthermore, microtubules dynamics of polymerization and depolymerization and the action of associated motor proteins [i.e. kinesin and dyneins (16, 17)] orchestrate the movement of pre-autophagosomal structures and autophagosomes across the cytoplasm during the process of autophagosome maturation (18, 19) and autolysosome bidirectional transport (20). The cooperation and competition between actin and microtubules are responsible for a large part of cellular mechanics. Together, these ATP-dependent cytoskeletal processes provide the mechanisms to overcome the energy barriers imposed by membrane elasticity and resistance to deformation that affect each step of the autophagic process (21). Finally, intermediate filaments (i.e. keratins and vimentin), which do not have evident dynamics and lack motor proteins, are thought to provide mechanical stability to the cell and its organelles (22). Intermediate filaments play a key role in autophagosome and lysosome positioning by providing a resistance to their free, unregulated movement (23). For instance, networks of vimentin cables have been observed to form cages around cellular organelles including the nucleus, endoplasmic reticulum, and mitochondria (24). Consistently with this regulatory function, pharmacological disruption of the vimentin network results in defective flow of the autophagic process (autophagic flux), the perinuclear position of autophagic vesicles and a loss of their region-specific localization at different stages of the process (23).

### Step-By-Step Mechanics of Autophagy

**Figure 1:**
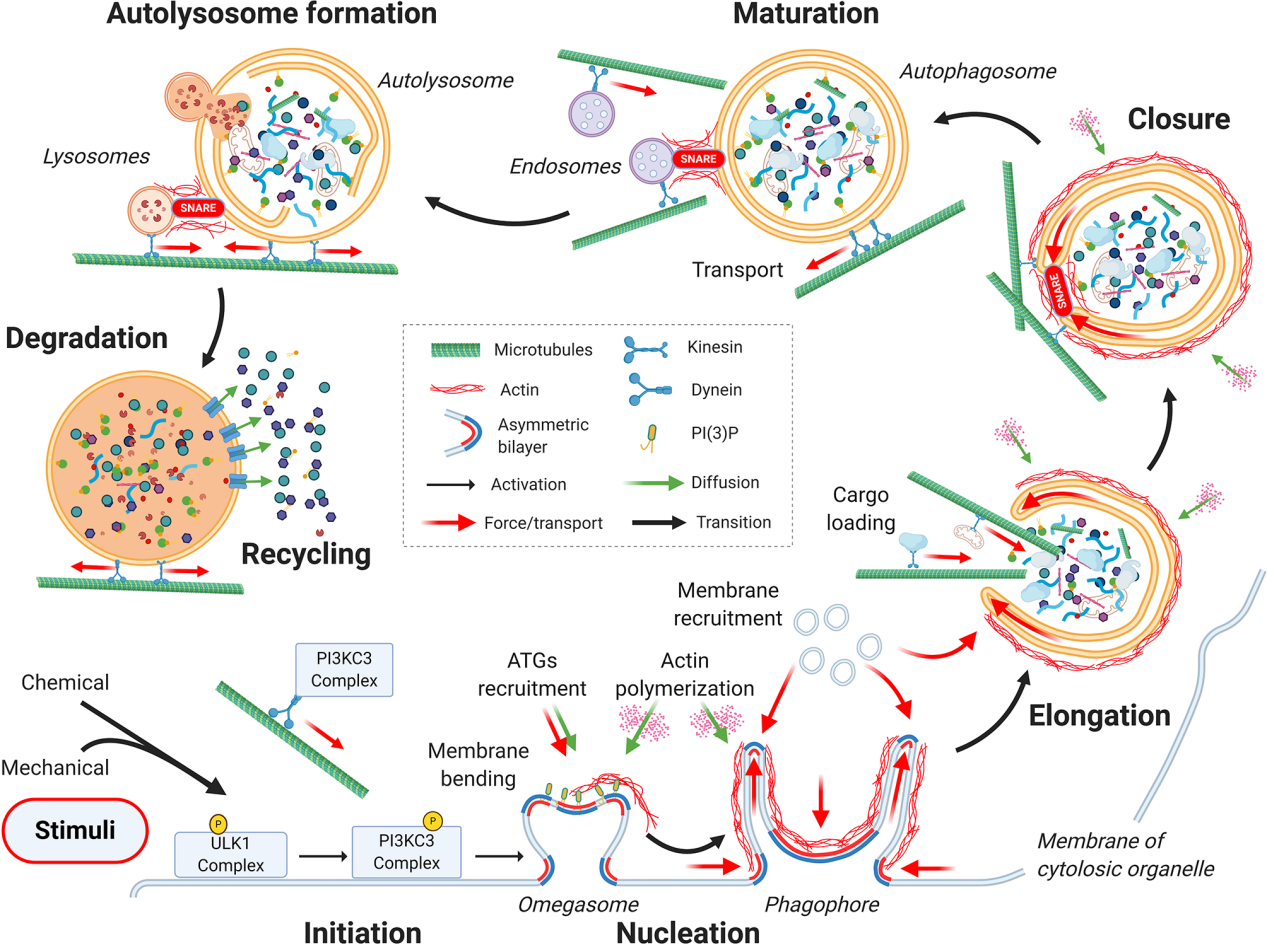
Mechanics of the autophagic process. From a mechanical point of view, the autophagic process can be divided into seven main stages: initiation, nucleation, elongation, closure, autophagosome maturation, autolysosome formation, and finally, cargo degradation and recycling. Cytoskeletal active processes and membrane organization during the sequential steps of autophagy are highlighted. See the main text for details.

From a mechanical point of view, the autophagic process can be divided into seven main stages, as depicted in **Figure 1**: initiation, nucleation, elongation, closure, autophagosome maturation and transportation toward the perinuclear region of the cell, fusion with the lysosome, and finally, cargo degradation and recycling (25).

### Initiation Stage

**Figure 2:**
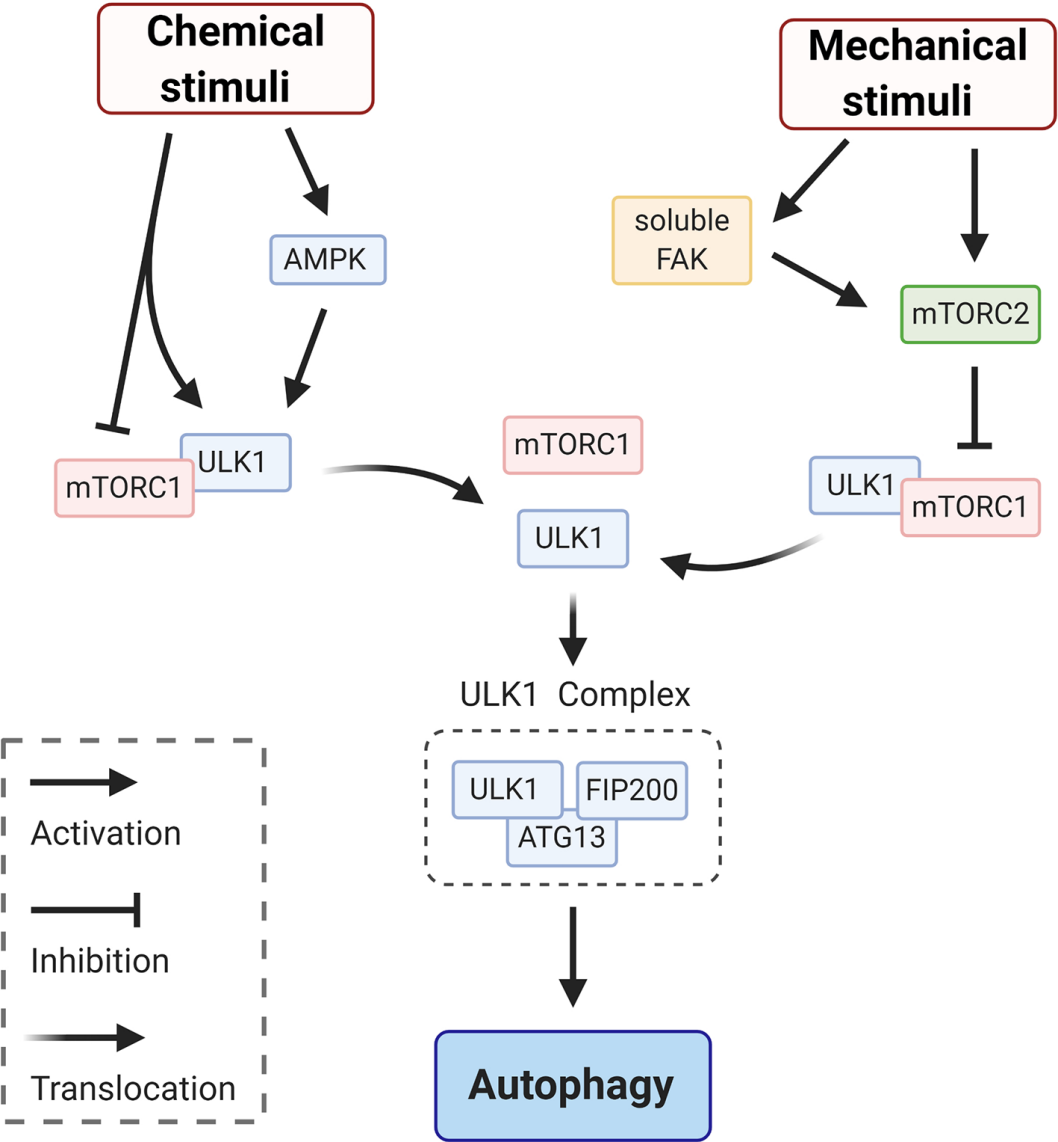
Schematic representation of signaling circuits for ULK1 complex activation in autophagy initiation. Initiation of autophagy *via* ULK1 signaling entails mTORC1 dissociation from ULK1, which becomes active and binds to ATG13 and FIP200 to form ULK1 complex. This dissociation and the following signaling cascade can be elicited by chemical stimuli, *via* e.g. enhancing the activity of AMPK and/or by mechanical stimuli. This second pathway is achieved *via* inhibition of mTORC1 by the mechanosensitive mTORC2, which responds to various mechanical stimuli. For instance, mTORC2 can be directly activated by the soluble form of focal adhesion kinase (FAK) that is releases from focal adhesion at low traction forces (e.g. detachment of the cells from the substrate).

Upon a chemical or mechanical stimulation the autophagic process begins, with the recruitment of core autophagy factors (**Figure 1**—initiation). This stage corresponds to the activation of the ULK1complex (26). As indicated in the schematic in **Figure 2**, modulation of the ULK1 complex is achieved by enhancing the activity of AMPK (induced by ATP depletion) (27) and/or by inhibition of the mechanistic target of rapamycin complex 1 (mTORC1) which acts as repressor of autophagy and, under basal conditions, maintains ULK1 in an inactive conformation (27). Canonical initiation of autophagy entails that metabolic stresses (chemical stimuli), such as nutrient deprivation, cause mTORC1 dissociation from ULK1, which becomes active and binds to ATG13 and FIP200 (ULK1 complex—**Figure 2**). This early signaling triggers the downstream events of autophagosome formation (**Figure 1**—initiation). Whether mechanical stresses and signals may play a direct role in ULK1 activation is still unclear. It has been reported that mechanosensitive (that responds to mechanical stimuli) mTORC2 (28, 29) is in a negative feedback loop with mTORC1 (30, 31), thus could indirectly induce activation of ULK1 to initiate autophagy *via* inactivation of mTORC1-repressor function (32). Importantly, mTORC2 can be mechanically activated by mechanosensitive, focal adhesion kinase (FAK) (33) (**Figure 2**). In adherent cells, FAK is part of focal adhesion, a protein complex mediating cell/substrate adhesion. Decrease of mechanical forces at the focal adhesions, which may occur upon detachment of the cells from the substrate or due to changes in rheological properties of the extracellular matrix, induce FAK dissociation from the focal adhesion complex (34). Soluble FAK is free to phosphorylate (activate) mTORC2 and consequently initiate autophagy (33). Interestingly, mTORC2 can also activate AKT, which reestablishes the inhibitory activity of mTORC1 through an indirect signaling cascade (28). Hence, FAK, mTORC2 and AKT may provide a possible negative modulation or an off-switch to detain the autophagic process (**Figure 2** **).**

### Nucleation

The process of formation of the initial complex of membranes that will elongate and mature into the autophagosome, begins with the binding of activated ULK1 complex to or in proximity of the sites for phagophore formation (35) (**Figure 1**—nucleation). In yeast, the phagophore assembly site is found in a dedicated and confined space between the endoplasmic reticulum (ER) exit site (36, 37) and the vacuole (yeast degradative lysosome) (38). Interestingly, in yeast, the autophagosome remains in this space throughout the autophagic process. Conversely, in mammalian cells, the phagophore assembly sites may be found at different cytoplasmic locations such as ER, ER-mitochondria junctions, or ER-plasma membrane, as well as specific subdomains of the plasma membrane containing the primary cilium (39–41). As consequence, the autophagosome needs to be transported throughout the cytosol for proper maturation. These differences between mammalian and yeast cells may reflect differences in structural complexity and spatial patterns of stress signal. It is tempting to speculate that the higher spatial complexity of the autophagic process seen in mammalian cells could be a result of higher mechanical complexity as compared to the wall-protected and sedentary yeast cells. The formation of the autophagosome begins with a curved membranous structure, named the omegasome for its shape resembling the Greek letter omega (42). The omegasome folds as a double membrane digit that receives lipids from most of the internal compartments of the cell (42, 43). The omegasome grows into a cup-shaped double membrane, known as phagophore or isolation membrane, which is typically connected with the ER membrane at its base (44). Eventually, the connection between the ER and the omegasome is sealed off and an independent double-membrane organelle is formed (42). To achieve this, several mechanical and energetic requirements need to be met. These include recruitment of specific ATGs, actin cytoskeleton to support and direct the curved membrane, and the recruitment of the necessary material, in particular phospholipids, to allow the *de novo* buildup of the phagophore (45) (see **Figure 1**—nucleation). ULK1 complex is responsible for the initiation all these mechanisms. As first step, ULK1 recruits and activates PI3KC3, a kinase complex formed by VPS34, Beclin-1, VPS15 and ATG14 (46). Activation of PI3KC3 occurs *via* ULK1 phosphorylation of Ambra1 (47), a Beclin-1 interacting protein. The PI3KC3 complex, which is tethered to the cytoskeleton through an interaction between the Ambra1 and dynein light chains (47), leads to PI3KC3 release from dynein light chain and the microtubule network, enabling the complex activation and translocation to the omegasome. In this location, PI3KC3 phosphorylates Phosphatidylinositol to generate Phosphatidylinositol 3-phosphate (PI(3)P), which promotes membrane bending and the recruitment of the additional ATG proteins required in the later stages of autophagosome formation (44) (see **Figure 1**—nucleation; ATGs recruitment). In addition to PI(3)P, membrane bending is also sustained by Atg17 (yeast counterpart of FIP200), a specific scaffolding protein that may also provide a curvature-sensing mechanism (48, 49). The Atg17 dimer has multiple hydrophobic residues that favor membrane interaction. Atg17 dimers arrange to tether the fused vesicles together, adopting a peculiar double-crescent shape (48) which is ideal to induce and sustain membrane bending. In addition, PI(3)P recruits specific membrane associated nucleation-promoting factors (NPFs), such as WHAMM (50), JMY (51), and WASH (52). In response to the specific localization of these factors, Arp2/3 and CapZ polymerize a network of branched actin proximal to the ER membrane (see **Figure 1**—nucleation; actin polymerization). This ATP-dependent and spatially controlled polymerization of actin generates pushing forces against the membrane, and thus sustaining the dome-shaped concavity therein (the isolation membrane) (45). In addition, this branched actin network provides a structural scaffold to sustain the pronounced curvature of the membranes forming the omegasome first and the subsequent phagophore. In particular, the latter would energetically tend to open into a spherical vacuole rather than keeping its typical cup shape due to the high curved edges. The preferred shape of a vesicle is defined by minimizing the membrane bending energy for a given enclosed volume (53). To overcome this energy barrier, cells take advantage of several tools such as asymmetric lipid and protein distribution between the two faces of the bilayer (e.g. PI(3)P and cholesterol) and the action scaffolding proteins (e.g. Atg17) and scaffolding cytoskeletal structures (actin) (53–56) (see **Figure 1**—nucleation). However, in a field that is disproportionally focused on protein-mediated signaling cascades, the importance of physical properties of the phospholipid bilayers has been largely overlooked. While PI(3)P and actin polymerization primes the physical environment, ULK1 also initiates a second crucial cascade leading to recruitment of phospholipids to assemble the pre-autophagosomal double membrane, which is achieved by the recruitment of vesicles receiving input from different membrane sources (mitochondria-associated ER membrane, ER, Golgi, plasma membrane, and recycling endosomes) (57, 58). This seems to be accomplished by two mechanisms: ATG9-vesicle transport and fusion with the omegasome (35) and ATG2-mediated transport of lipids from one donor compartment to the omegasome (59). Various signaling pathways such as EGF/Src induce incorporation and phosphorylation of cytosolic ATG9 in the target membrane and the formation of ATG9-vescicles (60, 61). The selectivity of the source of the membranes, depending on the type of autophagy and the nature of the cargo to be sequestered, is still debated (44). In general, intracellular membrane trafficking is regulated by the Rab family of small monomeric GTPases (62). In their GTP-bound form, Rab proteins recruit effectors to regulate vesicle trafficking, while hydrolysis of the bound GTP to GDP causes loss of effector binding and extraction from membranes. Upon activation of the autophagic process, activated Rab11/Ypt11 GTPase regulates the recruitment of ATG9 vesicles to the omegasome through the tethering of ATG9 to ULK1 (48, 49, 63). Actomyosin contractility seems to play a fundamental role in ATG9-vesicle transport. It has been shown that activation of myosin IIA *via* MLCK-like protein Sqa, which is downstream of UKL1, induces transport of ATG9 vesicles to the phagophore (64). While the proposed mechanism of cargo transport by myosin IIA seems farfetched, as myosin IIA is not a cargo transporter (65), it is possible that cables of actin under tension provide physical guidance for the flow of vesicles toward the phagophore. Recent work presents a different mechanism for the transport of phospholipids from the donor membrane to the forming autophagosome (66). Indeed, according to the experimental evidence, Atg9 establishes membrane contact sites with a donor compartment. Here phospholipids are transferred between compartments by lipid transfer proteins like Atg2, resulting in a net flow of lipids from the vesicles to the autophagosome without vesicle fusion (66).

### Elongation and Closure Stages

After priming of the physical environment, the membrane of the nascent phagophore elongates to an open cup-shaped structure thanks to the fusion of additional membrane (**Figure 1**—elongation). This novel structure encompasses a portion of the cell cytosol, which is ready to accept the material to be recycled (cargo loading) and finally seal through SNARE-mediated fusion (**Figure 1**—elongation and closure). In mechanical terms, the growth of the phagophore double membrane has the same mechanical requirements as the previous stage. Hence, this stage follows the same dynamics with lipid being recruited through ATG9-mediated fusion and/or transfer (**Figure 1**—elongation; membrane recruitment) and polymerization of actin cytoskeleton to structurally support the growing double membrane and maintain its shape (67) (**Figure 1**—elongation; actin polymerization). These processes are under strict regulation by several ATG proteins (such as ATG3, ATG7, ATG5, ATG12 and ATG16L1), including the lipidated LC3/GABARAP protein family (25). The lipidation process occurs by conjugating the cytosolic LC3-I protein to phosphatidylethanolamine (PE), which generates the membrane bound LC3-II (68–70). Importantly, LC3 lipidation requires the curved rim of the phagophore, as ATG3, the E2-like enzyme necessary for LC3 lipidation, only functions on a highly curved membrane (71). Additionally, it has been observed that the local curvature of the phagophore increases upon LC3 insertion, indicative of the curvature-inducing properties of LC3 (72). Hence, PE localization and enrichment on the phagophore inner membrane is fundamental for the progression of autophagy. Indeed, it has also been proposed that phospholipid transfer (PE precursors) from the ER to acceptor membrane on adjacent organelles (e.g. mitochondria) may be the mechanism which induces formation of the phagophore on sites other than the ER (73, 74). Among other cargo-receptors, LC3 is fundamental for selection and loading of specific cargo into the autophagosome [reviewed in (75)] (**Figure 1**—elongation; cargo loading). LC3 is also known to regulate cytoskeletal dynamics. On one hand, LC3 recruits NPFs (i.e. WHAMM and JMY) to promote the Arp2/3-mediated expansion of the membrane-proximal actin network and allow for the phagophore extension and shaping (13, 76). On the other hand, interaction of LC3 with microtubules has been proposed to mediate transport and selection of dysfunctional organelles (77), phagophore expansion and later in the process to mediate the closure of the autophagosome (16, 78, 79). Once loaded with its content, the phagophore closes into a double membrane organelle, the autophagosome proper, to confine its inner degradative space (80, 81) (**Figure 1**—closure). Prior to closure all the ATG proteins tethered to PI(3)P platform are removed from the surface of the autophagosome. This process requires the removal of PI(3)P by phosphoinositide phosphatases and possibly other factors (82–84). It must be noted that the clearance of PI(3)P is an important mechanism to dismantle the nucleating-elongating ATG machinery, required for the formation of the mature autophagosome (82). Finally, the closure of the phagophore is completed by a scission (or fission) process of the inner and outer membrane of the phagophore to generate the autophagosome with a double membrane (85). This process, still not completely understood, is mediated mainly by the endosomal sorting complex required for transport (ESCRT) (58, 86) and shares topology with canonical ESCRT-dependent cellular membrane scission processes, including cytokinesis, plasma membrane repair and multivesicular body biogenesis (87, 88). The ESCRT machinery is composed by distinct conserved complexes (ESCRT- I, -II and -II) and accessory proteins, such as ATPase protein VPS4, which disassembles and recycles ESCRT-III complex (89, 90). During the process, ESCRT-III subunits assemble into helical filaments providing the driving force to induce membrane deformation, while the recruitment of VPS4 drives membrane sealing (88) and, subsequent scission (91, 92). In addition to the ESCRT complex the motor protein Myo6 and the actin network participate in phagophore closure (87, 88). Altogether these components bring the open ends of the autophagosome in close contact to allow for SNARE-mediated fusion (93).

### Maturation and Formation of Autolysosome

Once the double membrane is fused, the process of autophagosome maturation begins. This requires fusion of the autophagosome with early/late endosomes (**Figure 1**—maturation) and transport towards a perinuclear region mediated by microtubules and dynein (**Figure 1**—maturation; transport) (18, 19). This is followed by fusion of the mature autophagosome with lysosome to form the autolysosome where the degradation of cargo occurs (**Figure 1**—autolysosome formation). Autophagosome–endosome/lysosome fusion may occur by a large variety of mechanisms, including kiss-and-run, complete fusions or fusion mediated through tubules (94). In these processes, docking and fusion appear to be two separately regulated events. Once the autophagosome and the lysosome encounter, the outer membrane of the autophagosome fuses with the lysosome forming an autolysosome. The fusion of endo-lysosomal vesicles with autophagosomes broadly requires Rab GTPases for trafficking and vesicles docking (in particular Rab7), membrane-tethering complexes and SNAREs to mediate vesicles fusion in a specific manner (95–97). The molecular mechanism regulating the fusion of autophagosomes with lysosomes has not yet been fully understood. Recent evidence shows that increased levels of PI(4)P on late endosomes/lysosomes stimulate the recruitment of the multisubunit homotypic fusion and vacuole protein sorting (HOPS) complex (98). HOPS complex, by interacting with LC3, tethers lysosomes to autophagosomes (99, 100) and, by direct interaction with autophagosome-localized STX17, facilitates the assembly of the SNARE complexes (101) between STX17 with its partners, ubiquitous SNAP29 and with lysosomal VAMP3 (95, 98). Beside the HOPS complex, TECPR1, a protein that localizes at lysosomal membranes has also been proposed as tethering factor that initiates autophagosome-lysosome fusion (102) by recruitment of LC3 matured autophagosomes to lysosomes and promoting the degradation of protein aggregates (103). The whole process of tethering and fusion is accompanied by the omnipresent actin network that stabilizes the curvatures and provides the mechanical energy to force the membranes of different organelles in close contact and fusion (11, 17). This latter process is mediated by WHAMM-dependent polymerization of branched actin network (cortactin and Arp2/3), leading to the appearance of stress-bearing actin comets (13), and by the unconventional myosin motor protein Myo1C (104). Contrary to the canonical, fast twitching myosins (e.g. Myosin IIA), Myo1C is a slow monomeric actin-based motor protein adapted for translocation of large loads at a slow pace. Though its mechanistic action is not completely elucidated, its suggested function is to link membrane cargo enriched in PI(3,5)P2 [produced by PIKfyve-dependent PI(3)P dissipation (105)] to the actin cytoskeleton (106) and to stabilize membrane ruffles (107).

### Cargo Degradation and Recycling

Upon fusion of lysosomes with the autophagosome and the degradation of the inner membrane, the process of autophagosomal cargo degradation begins, as depicted in **Figure 1** - Degradation and Recycling (43). During this step the autolysosomes significantly reduces in size (108), due to cargo degradation and the transport of small solutes (amino acids, monosaccharides and nucleosides) mediated by the solute carrier transporter (109). Solute transport across the autolysosome membrane is followed by the subsequent osmotic forces causing an outward flow of water. The shrinkage of the autolysosome is required for the following step of lysosome membrane recycling. The high membrane curvature, driven by the autolysosome shrinkage, recruits the protein complexes required for the processes of vesiculation and tubulation that allow lysosomes vesicles to reform (110). Autolysosome tubulation is also facilitated by the protein WHAMM, which, once recruited in autolysosome surface, promotes the formation of a branched actin scaffold that facilitates the process (108).

## Cellular Mechanics and Autophagy

**Figure 3:**
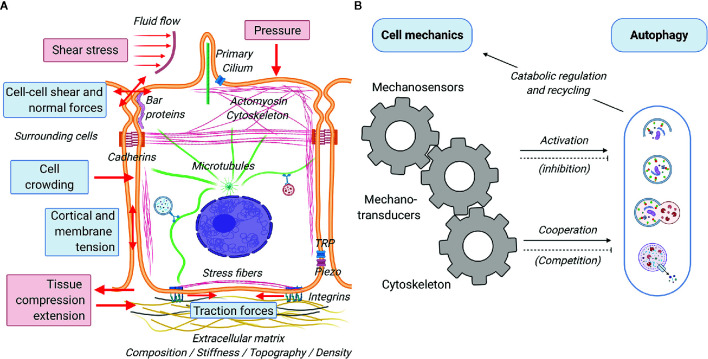
Schematic representation of cell mechanics and its interplay with autophagy. Cells subjected to a great variety of mechanical forces (red arrows) from the environment that generate cell-autonomous forces mediated by the cytoskeleton. **(A)** There are two main different categories of forces sensed by the cells: short scale (blue boxes) and large scale (red boxes). Short and long-scale forces are perceived by the cells *via* various mechanosensors, including interfacial protein complexes (integrin- and cadherin-mediated adhesions), mechanosensitive ion channels (TRP, piezo), tension and curvature sensors at the plasma membrane and actin cortex (BAR proteins, filamin), and the primary cilium. **(B)** Mechanical inputs are transduced to biochemical signals (mechanotransducers), such as Ca^2+^, transcription factors (YAP/TAZ) and signaling proteins (phosphatases and kinases) that affect the cytoskeleton, gene regulation, and other cellular functions. Autophagy is directly (cellular signaling mediated) and indirectly (cooperative action with the cytoskeleton) activated by mechanical processes. While likely to exist, negative feedbacks (inhibitions and competition for cytoskeletal elements) are still underexplored in the literature. Autophagy regulates various mechanical processes *via* ensuring recycling of cellular components and providing energy during catabolism.

A great variety of biophysical stimuli elicit cellular responses and determine cellular functions (**Figure 3A**) (111). Much of these stimuli stem from short-scale (**Figure 3A**, blue boxes) interaction between the cells and their physicochemical microenvironment. Cells have been reported to sense and respond to the a) physical status of the extracellular matrix (e.g. composition, stiffness, topography and density) by exerting traction forces on the substrate (112–114), b) geometrical cues (e.g. size, confinement, curvature) affecting cortical and membrane tension (115, 116), c) presence of surrounding cells (e.g. cell crowding) and their physical activity (e.g. pulling and pushing causing cell-cell shear and normal forces), and the chemical composition of the interstitial and luminal fluids (e.g. osmotic pressure inducing cell swelling or shrinkage and consequent variation in membrane tension) (117). In addition, cells are subject and respond to large-scale mechanical forces (**Figure 3A**, red boxes) such as shear stresses and fluid pressure due to flow of liquids or solid material in the lumen of tubular structures (e.g. gut, blood vessels and urinary tract), and lateral stretch and compression of tissues required for physiological function of lungs, muscles, and digestive system, among others (118–121). Short and long-scale force (summarized in **Figure 3A**) elicit reactive and adaptive cellular responses that primely involve active processes mediated by the cell cytoskeleton (4). This can be activated by the direct action of external mechanical cues on the cytoskeleton *via* sensing mechanisms involving various mechanosensors at the cell surface, such as mechanically activated ion channels (e.g. TRP and piezo), proteins sensing tension and curvature of the plasma membrane (e.g. BAR proteins) and of the cytoskeleton (e.g. filamin), and adhesion protein complexes (e.g. focal adhesion, adherens junctions) (122). These mechanosensors translate mechanical inputs into biochemical signals *via* mechanotransducers (AKA mechanotransduction process) that control cytoskeletal organization, membrane trafficking, gene expression profile and ultimately cellular function as a whole (4, 123, 124) (**Figure 3B**). Mechanosensing is generally achieved by a force-dependent conformational change of the sensing protein that may lead to the opening of a channel (typically calcium channels), which subsequently activates a cellular response *via* an electrochemical signal, or through the dissociation of proteins (mechanotransducer) from the sensing complex. In its freely diffusive form, the mechanotransducer participates in enzymatic reactions (e.g. phosphorylation), either as the enzyme or the substrate. As examples of both cases, the rise of calcium and/or the activation of protein phosphorylation cascades will lead to short- and long-term adaptation to mechanical stimuli. Acting as an essential part of the innate adaptive mechanisms of the cell, the autophagic response aids in the management of mechanical challenges and allows the cell to adapt to the everchanging physical environment (**Figure 3B**). In general terms, mechanical cues may affect the autophagic process in two ways: firstly, *via* specific crosstalk between mechanotransduction and autophagy regulatory proteins (e.g. mTORC, AMPK) (125, 126) responsible for the initiation and/or inhibition of the autophagic process and/or secondly, *via* the unspecific cooperation/competition mechanisms between mechanical processes and autophagy to recruit cytoskeletal elements and phospholipid membranes (127). A growing body of evidence demonstrates that indeed mechanical cues feed into the signaling required for the activation of autophagy (7, 128–130). Conversely, despite being highly plausible, the competition for cellular components between the two processes and the consequences of such, have still to be addressed by the literature. A final point of convergence is the regulatory role of autophagic catabolism and recycling of biological components in managing the turnover of cellular components necessary for proper execution of mechanical processes. In the following sections, we will discuss the crosstalk and interactions between cell mechanics and autophagy. Next, we will discuss in detail some the most relevant and better known connection between the mechanotransduction machinery and the autophagic process.

### Extracellular Matrix and Focal Adhesions

The macromolecular composition, structural architecture, and rheological properties of the extracellular matrix undergo constant remodeling due to the enzymatic and mechanical action of the cells (113, 131). These modifications and the remodeling processes deliver a versatile microniche that in turn affects cell phenotype and function and, when dysregulated, may lead to the emergence of disease states such as fibrosis and cancer (111). The ability of cells to sense mechanical properties of the extracellular matrix in normal and in pathological conditions can be attributed to the integrin-mediated adhesions, also known as focal adhesions (132, 133). Focal adhesions are composed of multiple mechanosensors (e.g., talin, vinculin), signaling molecules (e.g., FAK, Src, PI3K), adaptor proteins (e.g. paxillin) and actin linker proteins (e.g., filamin, alpha-actinin), which physically connect the integrins to the cytoskeleton [reviewed in (134)]. The binding of extracellular matrix ligands to integrin heterodimers promotes tension-induced conformational changes in the integrin cytoplasmic tail, leading to the recruitment of talin and paxillin (135, 136). As tension increases and focal adhesion mature, protein tyrosine kinase 2 and Src are recruited, providing the enzymatic kinase activity to promote downstream signal transduction, including Rho GTPase signaling, anoikis signaling, mitogenic signaling, and extracellular matrix turnover (137). Thus, integrin-mediated adhesions interact with the extracellular matrix and sense its rigidity, which in turn modulates cellular behavior including motility and migration (138). Several studies address how the extracellular matrix and integrin-mediated adhesion may trigger autophagy *via* FAK and ILK (integrin linked kinase), thus linking it to anoikis and cancer progression (detachment-induced cell death) (139–141). Importantly, these emerging interconnections between integrin-mediated adhesion pathways and autophagy are relevant for immunosurveillance (142) and thus impinge on the appearance of certain diseases, including cancer. Matrix constituents have been shown to regulate autophagy in both a positive (activators) or negative (inhibitors) manner. Decorin, collagen VI, kringle 5, perlecan, and endostatin function as activators (142–145), whereas laminin a2 is an inhibitor of the autophagic process (146). The extracellular matrix, which constitutes different physical and structural properties, can initiate biochemical signaling cascades that involve membrane receptors (e.g. integrins, VEGFR2, GRP78) (143, 144, 147), regulatory proteins (AKT, mTORC1 and 2) and autophagy specific effectors, including VPS34, Beclin-1 and lipidated LC3 (LC3-II) (142, 148). On the other hand, autophagy regulates integrin-mediated adhesion, and therefore cell migration, *via* controlling focal adhesion turnover through a mechanism involving LC3, paxillin and Src (149).

### Cell–Cell Adhesions

In addition to the extracellular matrix, cells in a tissue physically interact with other cells (e.g. epithelial cells, muscle cells) through transmembrane receptors that mediate extracellular bonds with receptors on neighboring cells to control tissue integrity and collective cell dynamics (150). Cell-cell contacts are mediated by various adhesion complexes, such as adherens junctions, tight junctions and desmosomes, each with distinct functions and molecular characteristics. Adherens junctions are force-sensor complexes. Tight junctions only appear to act in parallel to adherens junctions *via* a physical connection between the two complexes. The role of the desmosome in junctional mechanotransduction responses remains elusive. In adherens junctions, coupling between the cadherin transmembrane receptor and the actin cytoskeleton is mediated by a protein complex collectively termed the cadhesome network (151). Similarly to the previously described integrin-based adhesion, this complex has a well-defined spatial organization where force-transduction is mediated by protein conformation that in turn modulates the engagement of cadherins with the actin network (152, 153). Tension at adherens junctions induces an α-catenin conformational switch with consequent exposure of previously hidden binding sites for vinculin, resulting in increased functional integration of the complex with actin dynamics. Tension-induced conformation changes of vinculin can differentially engage the signaling layer to the actomyosin contractile machinery and enable localized actin polymerization through the Mena–VASP complex associated with vinculin (152, 153). Thus, vinculin serves the role of ‘molecular clutch’ that integrates mechanical and biochemical signals to engage and disengage the cell-cell junction to internal and external forces. This remarkable spatial organization and the molecular mechanism involved therein provide the cells with the strength and plasticity needed by the highly dynamic epithelial tissues during biological processes such as collective cell migration, wound healing, tissue stretching, etc. Autophagy plays a critical role in junctional homeostasis by actively regulating the recycling of the junctional complexes in response to various intra- and extra-cellular cues [reviewed in (154)]. Experimental evidence shows an autophagy-dependent translocation of cadherin (155) and claudin (156) from the cell membrane to the cytosol where they are subsequently degraded by the autophagosome or lysosome. The effect of cell-cell adhesion on the autophagic process has been less well studied. Nevertheless, it has been shown that the application of force to E-cadherin stimulates autophagy *via* Liver Kinase B1 (LKB1) activation, which recruits the autophagy-initiator-factor AMPK to the E-cadherin complex (157).

### Yes-Associated Protein/Transcriptional Co-Activator With PDZ-Binding Motif Signaling

In addition to what has been discussed in previous paragraphs, autophagy, and mechanosensing are interdependent *via* the YAP/TAZ system. Yes-associated protein (YAP) and the transcriptional co-activator with PDZ-binding Motif (TAZ) regulate gene expression in a force-dependent manner. Pioneering work of Piccolo and co-workers showed that mechanical forces regulate YAP/TAZ cytosolic localization and nuclear translocation (158). By analyzing YAP localization and transcriptional response, these investigators showed YAP activity to be regulated by extracellular matrix stiffness, cell density and cell geometry. When cells are at low density or on a stiff extracellular matrix, YAP and TAZ are active and localize in the nucleus, where they interact with the DNA-binding transcription factor TEAD to promote the expression of several growth-related genes and ultimately induce cell proliferation (159, 160). Conversely, when cells are at high-cell density or plated on soft matrix, YAP/TAZ are inactive in the cytoplasm (158, 161, 162) leading to contact inhibition of proliferation. This force-dependent control of proliferation is a fundamental mechanism to maintain tissue homeostasis and allow tissue repair. Impairment of this system may lead to uncontrolled cell growth (a cancer hallmark). Interestingly, one of the transcriptional targets of YAP/TAZ is Armus (163), a protein of the Rab-GAP family that mediates autophagosome-lysosome fusion (164). Consequently, it has been seen that the efficiency of the autophagic flux depends on the physical properties of the cell microenvironment *via* YAP/TAZ mechanical response (163). Furthermore, mTORC1 regulates YAP by mediating its autophagic degradation (165), further linking cellular nutrient status to YAP activity (166).

### Mechanosensitive Ion Channels

Calcium influx mediated by mechanosensitive channels have been implicated in the initiation and elongation stages of autophagy (167). ER-resident channels exhibit the potential to regulate autophagy at different stages (initiation as well as the autophagosome-lysosome fusion), due to its special role as a platform for autophagosome nucleation. However, these ER-resident channels have not being linked to mechanosensing. On the other hand, plasma membrane channels have been reported to control the initiation process *via* AMPK and mTOR. Interestingly two large families of calcium channels, the (osmo-mechano and voltage) transient receptor potential (TRP) channels and pore-forming Piezo (168) are known to be mechanosensitive. These two channel families are gated by changes in membrane tension, which may arise from stretch and compression of the plasma membrane during cell migration or when the cells are subject to shear flow. Similarly, these channels respond to osmotic challenges which increase membrane tension during cellular swelling (169).

### Membrane and Cytoskeletal Tensions

As a physical boundary between the cell and the environment, the plasma membrane constitutes a prime location for mechanosensation and mechanotransduction (170, 171). The poorly extensible lipid bilayer (rupture occur at only 3–5% area expansion) is mechanically supported by the actin cortex, which, thanks to its active dynamics, absorbs a great portion of applied stress, control folding and unfolding of plasma membrane into and out of membrane reservoirs and facilitates vesicle trafficking and fusion. Mechanical stimuli at the plasma membrane can be differentiated as tensile stress (cell stretching and hypoosmotic swelling), compressive stress (cell compression and hyperosmotic shrinkage), shear stress (flows of fluids over adherent cells) and forces generated by topographical cues (confinement caused by the physical microenvironment). Fluid shear stress has been reported to induce autophagy by activating the Rho GTPases (Rac1, RhoA, and Cdc42) with consequent upregulation of Beclin-1, ATG5, ATG7 and LC3 (172). Furthermore, cells respond to mechanical stress with rapid autophagosome formation through an mTOR-independent pathway (173). Autophagic response demonstrates high specificity to mechanical load with a transient and gradual response to the stimulus (half-maximal responses at ~0.2 kPa) (173). While the exact sensing and signaling mechanisms are not entirely clear, they may involve BAR proteins that have been identified as primary membrane tension sensors (174). Another mechanism of tension sensing involves the actin scaffold protein filamin. Filamin A control the tensional state of the actin cytoskeleton by mediating crosslinking of actin filaments at large angles (175, 176). When cells are challenged by sheer flow, filamin accumulates throughout the cell, increasing the overall mechanical stability of the cytoskeleton (177). In addition, filamin A crosslinks integrin with actin and thus mediates force-dependent reinforcement at the focal adhesions (178). In response to tension, filamin A undergoes conformational changes that promote its ubiquitination and subsequent targeting by chaperone-mediated autophagy (179).

### Mechanosensing at the Primary Cilium

Key processes such as cell migration, differentiation, cell cycle re-entry and apoptosis largely depend on the specific activity of the primary cilium (180). Found in the majority of cell types, the primary cilium is a non-motile microtubule-based appendix that senses extracellular chemical and mechanical stimuli (181, 182). For instance, the cilium in kidney cells is a flow sensor. Sheer forces causing bending of the the cilium induce calcium entry into the cell *via* polycystin-2 (PC2) and transient receptor potential vanilloid 4 (TRPV4) (183). This sheer-stress-dependent signaling triggers autophagy and leads to cell size regulation (184, 185) through the LKB1-AMPK-mTOR signaling pathway (186). In contrast to starvation-induced autophagy, mechanical signaling from the cilium initiates autophagy in a ULK1, Beclin-1 and PI3K/VPS34 independent manner (187, 188). It was recently reported that the PI3KC2α lipid kinase (PI3K class II), required for ciliogenesis and cilium function, can promote the synthesis of a local pool of PI(3)P in response to shear stress (188). In turn PI(3)P is crucial for Rab11a membrane mobilization and activation (189, 190), and serves as platform for autophagosome assembly and formation (191, 192). On the other hand, primary cilium length and assembly (ciliogenesis) are modulated by autophagy. This involves the degradation of ciliogenesis regulators (193, 194), as evidenced that during starvation, several components of the autophagic machinery (including ATG16L1) localize at the cilium’s basal body (40).

## Mechanics of Autophagy During Cancer Transformation

**Figure 4:**
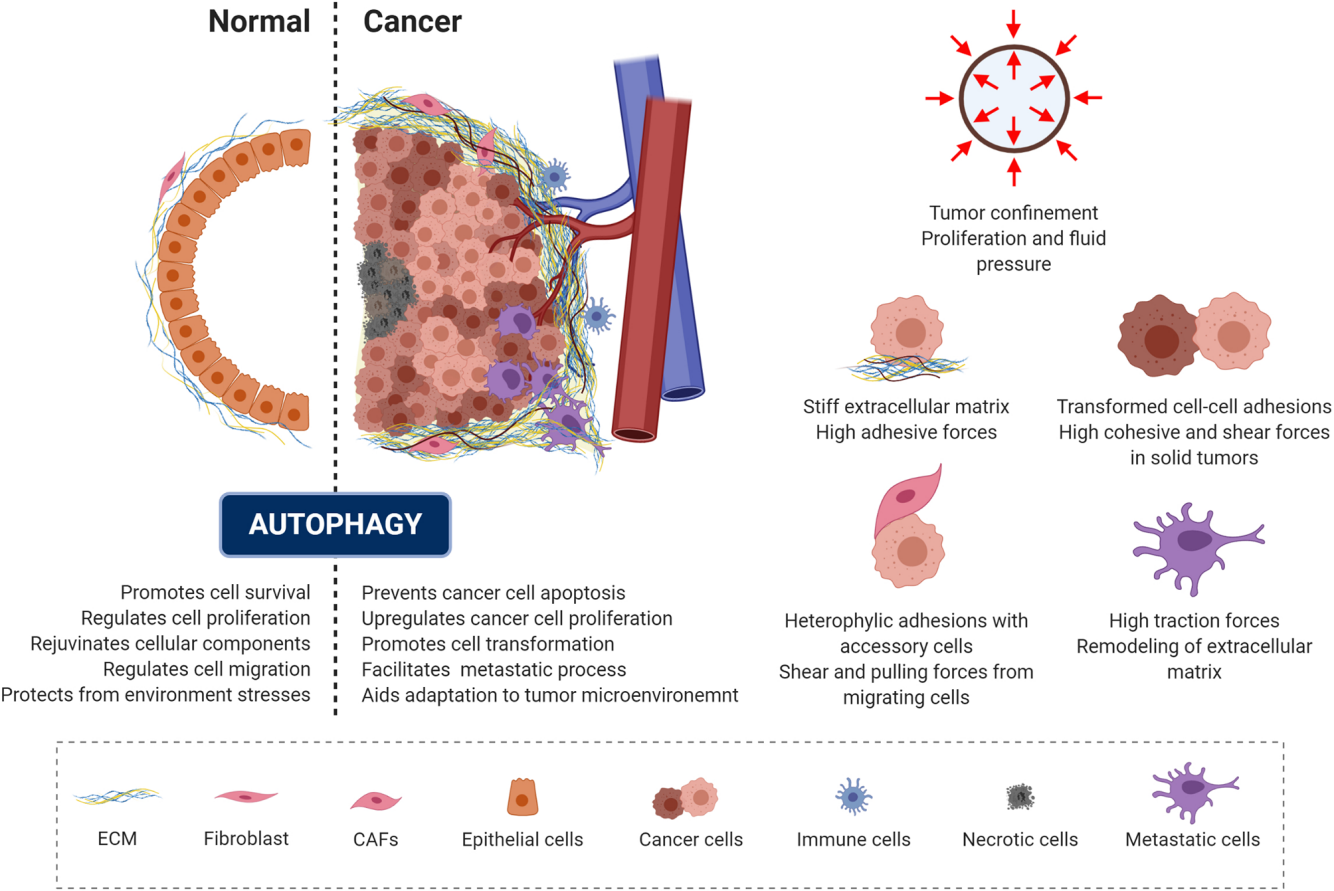
Autophagy and mechanics during cancer transformation. The role of autophagy in preserving cell homeostasis and protecting cells from mechanical environmental stress is represented and described in the left panel (dotted line separates the normal and cancer context). Cancer cells exploit autophagy to adapt to the tumor microenvironment and promote malignant progression. Right panel illustrate the mechanical components of the tumor microenvironment.

Malignant transformation is accompanied by a progressive loss of tissue homeostasis and perturbations of tissue architecture. It is widely recognized that a critical component of this transformation involves alterations in the mechanical phenotype of the cell and of the surrounding microenvironment, creating a peculiar mechanical milieu predominantly composed of cancer cells surrounded by a dense extracellular matrix (6, 195, 196). In addition, a set of accessory cells may be found in the tumor microenvironment, including blood and lymphatic vascular cells, lymphocytes, inflammatory cells and cancer associated fibroblasts (6, 195, 197). Depending on the context and stage of cancer development, autophagy has been recognized as a “double-edged sword” (**Figure 4**, left panel), as it can act as a mechanism for either tumor-suppression or tumor-promotion depending on the cellular context in which it acts (198, 199). Consistent with its role in promoting cell survival and rejuvenating cellular components, autophagy serves as a quality-control mechanism, detecting changes in organelle architecture and protein folding and thus preventing tumor initiation. On the other hand, these same mechanisms promote cancer cell survival and escape from apoptosis. This occurs by aiding the responses against environmental stress and generating the energy needed for unregulated growth and metastasis through the recycling and degradation of cellular organelles (**Figure 4**) (198, 200–202).

In the context of solid tumors, several mechanical aspects of the tumor microenvironment contribute to the tumor-promoting function of autophagy (**Figure 4**, right panel). When confined by the extracellular matrix, cancer spheroids experience forces exerted by the expanding tumor mass as a result of unchecked proliferation and the resistance to deformation of the surrounding stromal tissue (203, 204). This causes increased interstitial pressure (203) and generates shear stress within the tumor microenvironment (7, 205, 206). Eventually, this mechanical stress affect cell growth directly, by compressing cancer cells, and/or indirectly, by compressing the surrounding blood and lymphatic vessels (207). Due to the sustained compression of the vasculature within the tumor, poor tissue perfusion causes hypoxia and eventually necrosis within the tumor (208). Hypoxia promotes epithelial to mesenchymal transition (EMT), a reorganization of the cytoskeleton and dissolution of the epithelial cell-cell junctions. This enables dynamic cell elongation, directional motility (209), and consequently an increase in the metastatic potential of the tumor cells. Furthermore, during EMT the composition of intermediate filaments changes, switching from keratin to vimentin (195, 210). Furthermore, during this process the actin cytoskeleton becomes hypercontractile through the TGF-β-dependent activation of pathways such as Rho GTPases, p38MAPK and ERK1/2 (211). This pathway activation triggers actin reorganization and formation of cellular protrusions, including lamellipodia and filopodia (212, 213). Furthermore, TGF-β and hypoxia also promote the formation of cancer associated fibroblasts (214, 215), which interact with each cellular component of the tumor microenvironment.

By mediating extracellular matrix stiffness, the cancer associated fibroblasts can regulate the cancer cell cytoskeleton (216–218). These changes to the cellular cytoskeleton during transformation or EMT drastically alters their mechanical phenotype and in particular the degree of tension exerted on neighboring cells and the extracellular matrix, leading to increased migration, invasion and dissemination potential (201, 219). To successfully metastasize, tumor cells migrate locally and invade into surrounding tissue to gain vasculature access, and subsequently intravasate through the basal membrane and detach from the extracellular matrix to become circulating tumor cells (220, 221). Ultimately, circulating tumor cells that survive in circulation can extravasate from the bloodstream and engraft in secondary tissue sites and thus forming metastatic foci (222). All cells that travel in the bloodstream experience fluctuating levels of shear stress. Hemodynamic shear stress, caused by the movement of blood along the cell surface, is influenced by both the fluid viscosity and fluid flow velocity (210). Shear stress can also be caused by frictional interaction with endothelial cells (6, 223). Equally, tumor cells within the bloodstream must survive harsh conditions, including extracellular matrix detachment-induced apoptosis (i.e. anoikis), immune system assault, along with the variations in shear stress (222). The physiological shear stress (0.5–3 Pa) caused by blood flow may suppress cancer cell proliferation but may also promote migration and adhesion (224–227). Substantial evidence suggests that mechanical stress, such as compressive and shear forces, in the tumor milieu boosts malignant progression by inducing autophagy (129, 130, 172, 228). Consistent with this, cervical cancer cells exposed to pulses of laminar shear stress of 2 Pa (over 3 and 6 minutes) undergo autophagy, by a lipid raft-mediated p38MAPK dependent process, and delay apoptotic cell death (130). However, shear stress is not necessarily beneficial to the cancer cell. Conversely, elevated levels of shear stress (6 Pa), as occurs during intense exercise, has been shown to promote tumor cell death (229). Furthermore, fluid shear stress in the range of 0.05 to 1.2 Pa is shown to trigger cancer cell death through apoptosis and autophagy in several cancer cell lines, including hepatocarcinoma, osteosarcoma, oral squamous carcinoma, and carcinomic alveolar basal epithelia. Interestingly, this fluid shear stress induced death did not occur in non-cancerous cells (230). Thus, it appears that depending on both the intensity and the duration of the shear stress, autophagy may act as either a pro- or anti-survival mechanism. Furthermore, it has been shown that autophagy induced by shear or compressive stress plays a role in cytoskeletal remodeling and in the recycling of proteins essential for cancer progression (149, 172). Indeed, increased tissue stiffness is implicated in the control of several tumor features, such as growth, invasion, and metastasis (203, 231, 232). Accordingly, it has been observed that the stiffness of cancerous tissue of breast, hepatic and liver origin is higher than that of the corresponding respective physiological context (233–236). Extracellular matrix stiffening in tumors is produced as consequence of stroma reorganization, through the excessive activity of extracellular matrix proteins and enzymes that covalently cross-link collagen fibers and other extracellular matrix components (237, 238). Collagen crosslinking enhances integrin activation, focal adhesion maturation, intracellular contraction and thus causes a subsequent increase in the stiffness of the actin cytoskeleton, which may favor cancer cell migration and invasion (214, 239–241). The higher extracellular matrix stiffness also plays a role in the onset of the malignant phenotype: cytoskeletal tension leads to increased cell- extracellular matrix adhesions and disruption of cell-cell junctions (242). Enhanced collagen deposition in the extracellular matrix leads to activation of the Hippo signaling pathway (159, 162, 243) with a consequent loss of contact inhibition. Autophagy is also reported to have a pivotal role at the center of these processes. Autophagy is reported to be compromised in contact-inhibited cells in both 2D or 3D-soft extracellular matrix cultures. In such cells, YAP/TAZ (previously mentioned to be regulated by mechanical forces) fail to co-transcriptionally regulate the expression of myosin-II genes, resulting in the loss of F-actin stress fibers, which leads to impairment in autophagosome formation. This loss of F-actin stress fibers is also associated with a reduction in the number of ATG16L1 puncta per cell and with decreased co-localization of ATG9A-LC3, suggesting an alteration in the trafficking of key autophagy proteins and thus a defective autophagic response (244). Furthermore, compressive stress-induced autophagy can promote secretion of matrix metalloproteinase-2 and the turnover of the focal adhesion paxillin, boosting the invasiveness of the HeLa cervical cancer cell line (129). In line with these results, it has been suggested that paxillin binds directly to LC3 to stimulate focal adhesion disassembly in MDA-MB-231 human breast cancer and in B16.F10 mouse melanoma cell lines, and furthermore promote metastasis *in vivo* in the 4T1 mouse mammary tumor model (149). Another mechanism of force sensing in cancer involves filamin A. This actin and actin-integrins crosslinker is down-regulated in human bladder cancer, reducing autophagy in these cancer cells, as indicated by the decrease in the levels of LC3-II and decrease in LC3-I (245). It has been further reported that upon overexpression, filamin A attenuates autophagy and suppresses the invasive ability in cancer cells. The mechanism of action may involve the inhibition of matrix metalloproteinases expression, regulation of integrin function and enhances apoptosis (245–247). Interestingly, YAP/TAZ signaling has been shown to stimulate filamin A transcription to maintain actin anchoring and crosslinking under mechanical tension (248). This could be a potential mechanism for cancer cells, to control autophagy through a crosstalk between YAP/TAZ and cytoskeletal elements. Low mechanical stress has been shown to activate Caveolin-1, triggering the FAK/Src and ROCK/p-MLC pathways, which are involved in the reorganization of the cytoskeleton, cell motility, focal adhesion dynamics and breast cancer cell adhesion (227). PI3K/AKT activation and β-Catenin-TCF/LEF-dependent activity downstream from Caveolin-1 also correlates to increased VEGF expression and thus greater angiogenic potential of tumor (249). Shear stress-induced Caveolin-1 activation can induce PI3K/AKT/mTOR signaling and metalloprotease activity, which have been shown to promote cell motility and metastasis of breast carcinoma cells (250). Conversely, it was determined that phosphorylated Caveolin-1 functions to activate autophagy through binding to the Beclin-1/VPS34 complex under oxidative stress and to protect against ischemic damage (251). These data suggest that Caveolin-1 function might be cell-context dependent (252), resulting in different autophagic outcomes. Interestingly, similarly to autophagy, Caveolin-1 has also been implicated both in tumor suppression and progression (253, 254). Although potentially protective in bourgeoning tumors, higher levels of either Caveolin-1 mRNA or protein have been reported in varying cancers strongly correlating with poor survival in advanced cancer patients (227). This further implies that Caveolin-1 has a role in the metastatic process, as evidenced by increased migration, invasion and anchorage-independent growth (255). Furthermore, recent studies have unveiled the existence of an interplay between the primary cilium and autophagy in the regulation of cancer development and progression (256–258). In addition to being considered as a survival mechanism in tumorigenesis, excessive accumulation of autophagosomes may induce autophagic cell death or apoptosis (259–262), which, in the context of cancer, limits tumor growth and spread. Recently, Wang and collaborators showed that acute shear stress (10 Pa for 60 min) promotes autophagosome accumulation, which is accompanied by increased fusion of autophagic vesicles with multivesicular bodies, and reduction of autophagosome-lysosome fusion, in HeLa and MDA-MB-231 cell lines (263). Furthermore, the inhibition of autophagosome degradation, induced by mechanical stress, is associated with increased release of autophagic components in extracellular nanovesicles, possibly through a Ca^2+^-dependent pathway involving autophagy, multivesicular bodies and exosomes (263). Thus, exosome secretion might provide a supplementary pathway to maintain cellular homeostasis when the autophagy pathway is damaged or insufficient to degrade large amounts of damaged proteins and prevent cell death (263). In conditions of mechanical stress, these results suggest a possible crosstalk between degradative and secretory autophagy to maintain cellular homeostasis and tumor cell survival (264). Furthermore, following pathological stress, harmful nucleic acids, molecular chaperones, cytosolic proteins, and misfolded proteins are released into the extracellular space through exosomes and may contribute to tumor progression and metastasis (265, 266).

As we have highlighted in the previous sections, the relation between cell mechanics and autophagy goes two ways. In the context of cancer, autophagy regulates multiple metastasis-related signaling pathways associated with cell mechanics depending on cell type and tumor microenvironment. Autophagic protein LC3-II mediates the targeted degradation of focal adhesion proteins such as Src and paxillin (149, 267) to promote focal adhesion disassembly and turnover and lead to cell migration. Furthermore, integrins can be differently recycled and degraded, depending on their conformation, activation by ECM proteins, and binding of effector proteins, such as TLNs and FERMTs/kindlins. Integrins trafficking, recycling and degradation affect their availability at the plasma membrane, focal adhesion dynamics and Rho GTPase-mediated cytoskeleton remodeling to facilitate cell motility (127, 268). While nutrient starvation (269) increases integrin internalization and ECM degradation (270), hypoxia (271) promotes recycling of specific integrin. Since nutrient starvation and hypoxia are both hallmarks of tumor microenvironment, further investigation into how autophagy regulates integrin trafficking may provide insight into the overall role of autophagy in cancer metastasis and lead to the understanding of how microenvironmental stress act on cell mechanics to induce cancer cell exit from the primary tumor.

## Mechanobiology of Autophagy in Cancer Treatment and in Avoidance of Chemoresistance

In the last decade a plethora of new treatments has been introduced that significantly improved the survival of cancer patients. Despite this, highly aggressive cancers often develop primary or acquired resistance that finally cannot be treated. To this aim, new therapeutic approaches are required to overcome drug resistance and improve treatment response. Based on the reviewed literature, considering the “mechanobiology of autophagy” might represent a novel and promising approach. Indeed, even if, to the best of our knowledge, to date there are no drugs approved by the FDA or currently being investigated in clinical trials that consider the mechanobiology of autophagy in their approach there are examples of proteins/pathways that are modulated by mechanical forces thus affecting autophagy.

As previously mentioned, a pathway that is activated in cancer cells, which is regulated by mechanical forces, is the Hippo–YAP/TAZ pathway, whose inhibition has been shown promising results in reducing therapy resistance [for recent reviews see (272, 273)]. Interestingly, blockade of this pathway also reduces autophagy (244), which is targeted by several drugs currently under investigation in clinical trials, suggesting that dual inhibition of YAP-TAZ pathway and autophagy could improve treatment response. Mechanics also regulate epidermal growth factor receptor (EGFR), a protein that is regularly amplified or mutated in glioblastomas, and where autophagy is enhanced promoting cell survival (274). Inhibition of autophagy, in addition to radiotherapy, already showed positive results which might be further improved by considering the mechanics of cancer. Consistently, inhibition of Janus-associated kinase (JAK) by Ruxolitinib, a drug currently used in myeloproliferative neoplasms which inhibits cell contractility, preventing signaling downstream of focal adhesions, has recently been shown to induce autophagy (275), thus a combination of ruxolitinib with pharmacological inhibitors of autophagy needs to be followed for cancer treatment. Another drug that is currently being studied in clinical trials is losartan, an angiotensin II receptor blocker, which reduces intratumoral interstitial fluid pressure in solid tumors (276). Interestingly, this drug has also been shown to inhibit autophagy promoting autophagic cell death in cancer cells (277), again confirming the importance of targeting autophagy and mechanobiology in cancers.

## Concluding Remarks

In recent years, autophagy has emerged as one of the key regulators of cellular, tissue, and organism homeostasis. Vibrant research in this field has brought to light the intricacies of autophagy’s molecular machinery, together with its biochemical regulation and biomedical consequences associated with its impairment. The complex mechanobiology regulating cellular mechanical and biochemical processes is also a bourgeoning field. In this review, we hoped to bring to light the role of physical forces in autophagy regulation and their potential implications in both physiological as well as pathological conditions. More importantly, we hoped to raise questions to help investigate the mechanical requirements of autophagy and appreciate the extent to which mechanical signals affect this process. For instance, a diet rich in saturated fatty acids can negatively impact autophagic flux in neurons (278, 279). Interestingly, as the steric conformation of these phospholipids is known to mechanically decrease membrane bending, it could consequently impair autophagy by preventing vesicle fusion. However, the mechanical role of phospholipids is largely overlooked in the literature and it could represent an important area for future investigation. Similarly, to provide new frontiers for exploration, areas worthy of investigation are the action of cytoskeletal dynamics, the mechanical interplay between cellular processes, and the role of environmental cues. To achieve this a paradigm shift is required, one that adopts modern interdisciplinary approaches combining cell biology, physics, and engineering (280). To this end, cutting-edge techniques such as superresolution microscopy and the control of the mechanochemical environment (281) (e.g. by incorporating biomimetic substrates and microfluidics) will open exciting opportunities and perspectives. Combined, these technological and conceptual new directions will lead to a better understanding of autophagy and mechanisms onsetting related diseases, which in turn would pave the way to the identification of new pharmacological targets.

## Author Contributions

MH-C, LM, PL, AR, and CB prepared the figures. JP and FP investigated the intersection of autophagy and cell mechanics in the literature by data mining (Cytoscape). MH-C, LM, JP, FP, PL, PA, GO, EM, AR, and CB reviewed the literature. MH-C, LM, GO, EM, AC, AR, and CB wrote the manuscript. GO, EM, AR, and CB proofed the manuscript. AR and CB conceptualized and supervised the work. All authors contributed to the article and approved the submitted version.

## Funding

AR and CB are supported by ANID PIA192015; EM and AC by ANID PIA 172066; GO by CONICYT FONDAP-15130011, IMII P09/016-F, and FONDECYT 1180241; EM by FONDECYT 1200499; AC by FONDECYT 1171075 and FONDAP no 15130011. AR acknowledges PUENTE 004/2019 and 012/2020 from the Pontificia Universidad Católica de Chile. LM, PL, JP, and FP acknowledge IPREint20 from the Pontificia Universidad Católica de Chile.

## Conflict of Interest

The authors declare that the research was conducted in the absence of any commercial or financial relationships that could be construed as a potential conflict of interest.

## Abbreviations

AMPK, AMP-activated protein kinase; ATG, autophagy related protein; EGF, epidermal growth fctor; EMT, epithelial to mesenchymal transition; ER, endoplasmic reticulum; ESCRT, endosomal sorting complex required for transport; FAK, focal adhesion kinase; GABARAP, gamma-aminobutyric acid receptor-associated protein; JMY, junction-mediating and regulatory protein; LC3, microtubule-associated protein 1A/1B-light chain 3; LKB1, liver kinase B1; MLCK, myosin light-chain kinase; mTOR, mechanistic target of rapamycin; NPFs, nucleation-promoting factors; PE, phosphatidylethanolamine; PI(3)P, phosphatidylinositol 3-phosphate; PI3KC3: class III phosphatidylinositol 3-kinase; SNARE, soluble NSF attachment protein receptor; TAZ, transcriptional co-activator with PDZ-binding motif; TRP, transient receptor potential; VEFGR2, vascular endothelial growth factor receptor 2; VPS, vacuolar sorting protein; WASH, WAS protein family homolog; WHAMM, WASP homolog associated with actin, membranes and microtubules; YAP, Yes-associated protein; CAFs, cancer associated fibroblasts; ECM, extracellular matrix; FIP200, focal adhesion kinase family interacting protein of 200 kDa; ULK1, unc-51-like kinase 1.
